# Acute bilateral uveitis and right macular edema induced by a single infusion of zoledronic acid for the treatment of postmenopausal osteoporosis as a substitution for oral alendronate: a case report

**DOI:** 10.1186/s12891-016-0926-x

**Published:** 2016-02-11

**Authors:** Yiming Tian, Rui Wang, Lianyuan Liu, Chunming Ma, Qiang Lu, Fuzai Yin

**Affiliations:** Department of Endocrinology, The First Hospital of Qinhuangdao, Hebei, China; Liaison Office, the First Hospital of Qinhuangdao, Hebei, China

**Keywords:** Zoledronic acid, Alendronate, Bisphosphonate, Uveitis, Osteoporosis, Macular edema, Acute phase reaction

## Abstract

**Background:**

Zoledronic acid-induced uveitis (ZAIU) is rare but severe, and has been recently considered part of an acute phase reaction. Only 15 cases have been reported since 2005. Here we describe a case with macular edema, which is the first reported case observed after long-term alendronate tolerance.

**Case presentation:**

A 63-year-old Asian woman received her first intravenous zoledronic acid treatment for the management of postmenopausal osteoporosis as a more convenient substitute for oral alendronate. Twenty-four hours later, bilateral eye irritations, periorbital swelling, blurred vision, and diplopia presented. The complete blood count and transaminase levels were normal, but the erythrocytic sedimentation, C-reactive protein, and serum C4 levels were elevated. On detailed ophthalmological examination, a diagnosis of bilateral acute uveitis and macular edema in the right eye was made. The ocular symptoms were not improved until administration of topical and oral steroids. Complete resolution was achieved. There was no rechallenge of bisphosphonates, and no recurrence at 6 months follow-up. Based on an extensive review, abnormal fundus is rarely reported, especially in cases of macular edema. Rechallenge with zoledronic acid in five cases induced no additional uveitis, and changing the medication to pamidronate in another patient was also tolerated. Interestingly, our patient suffered from uveitis soon after intravenous zoledronate exposure after a two-year tolerance to oral alendronate.

**Conclusions:**

This is the first report of zoledronic acid induced uveitis with macular edema after long-term alendronate tolerance. Prior oral alendronate may not entirely prevent ZAIU. Steroids are usually necessary in the treatment of ZAIU. Bisphosphonate rechallenge is not fully contraindicated, and prior steroid administration may be a more reasonable treatment choice according to the available evidence.

## Background

Zoledronic acid is generally well-tolerated in the management of osteoporosis and other metabolic bone diseases [1]. The most frequent adverse event is acute phase reaction (APR), which occurs in nearly half of the patients after zoledronate infusion, but usually lasts for a short time with less severity [2]. Zoledronic acid-induced uveitis (ZAIU) is rare; the incidence was reported to be 0.8–1.1 % [3, 4]. Some severe cases have presented with transient reduced visual acuity [5–14]. Previously, other bisphosphonates (pamidronate [15, 16], alendronate [17], and clodronate [18]) have also been reported to be associated with acute uveitis. Uveitis is not commonly considered to be part of the APR, but they occur within the same time frame, suggesting that it may have a similar pathogenesis [2, 19, 20]. Stepwise regression of a phase three clinical trial showed less APR after zoledronate infusion in previous users of oral bisphosphonates [21].

Due to the low incidence [3, 4], even a prospective randomized study failed to document the risk factors of ZAIU [3]. Presently, only 15 individual cases have been reported [5–14, 22–25]. We describe a case accompanied with right macular edema occurring during the management of postmenopausal osteoporosis with the substitution of the more convenient orally administered alendronate. To our knowledge, this is the first report of ZAIU with macular edema, as well as the first report of ZAIU after long-term alendronate tolerance.

## Case presentation

### Case report

A 63-year-old Asian woman received her first dose of zoledronic acid (Aclasta®, 5 mg/100 mL solution; Novartis, San Mateo, CA, USA) for postmenopausal osteoporosis. She suffered from a past hepatitis B virus infection. In the past 2 years, she was on oral alendronate (70 mg, once per week), and the patient regularly used calcium and vitamin D supplements. Slit-lamp and fundoscopy examinations before the application of zoledronic acid revealed right epiretinal membrane and bilateral xerophthalmia. Twenty-four hours after the infusion (5 mg in 100 mL normal saline over 15 min at a constant infusion rate), she began experiencing multiple symptoms, including fatigue and weakness, generalized aches and pains, bilateral eye irritation, periorbital swelling, blurred vision, and diplopia.

**Fig. 1:**
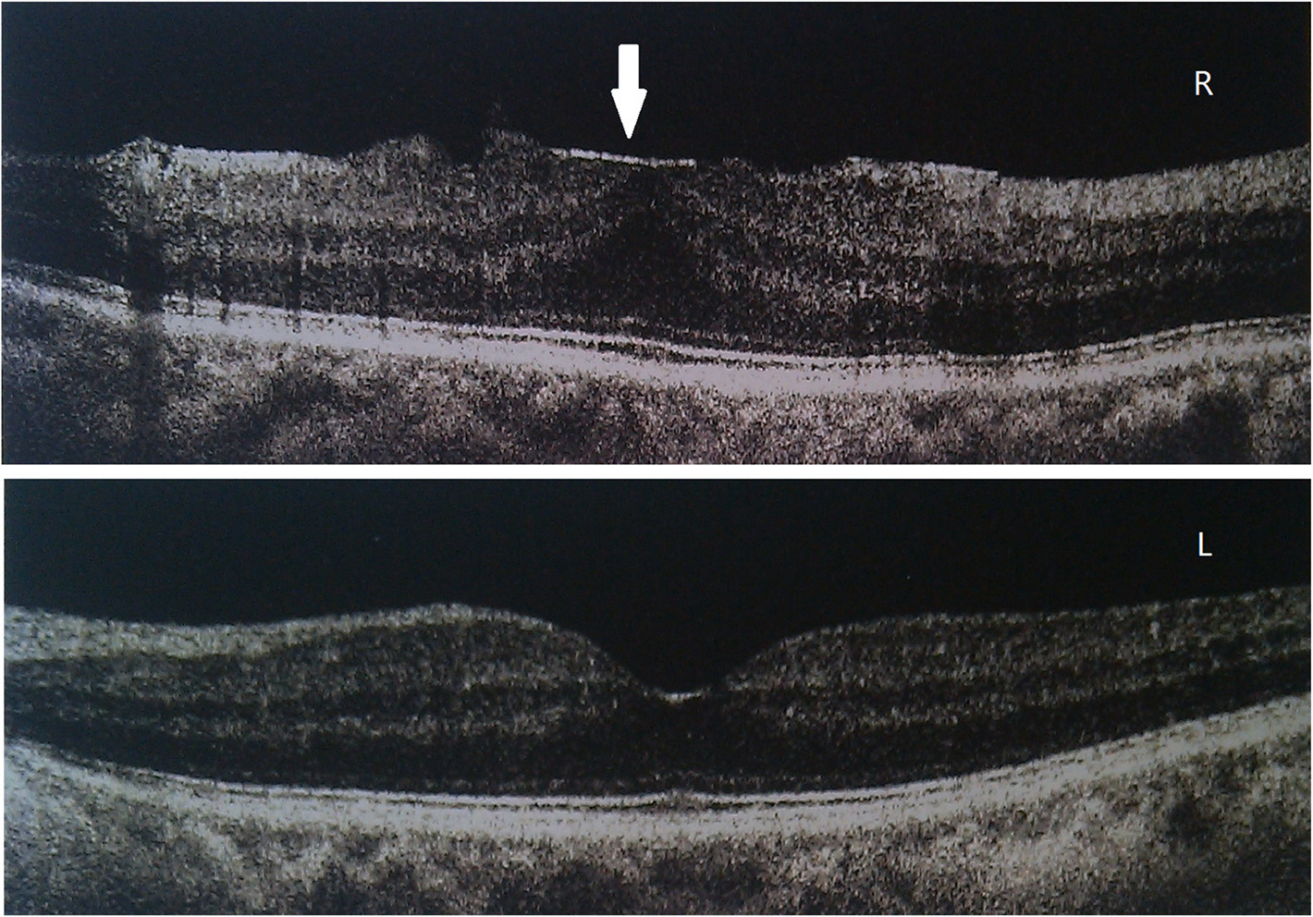
Optical coherence tomography examinations revealed right macular edema in a postmenopausal women with ZAIU. Two days after the intravenous administration of zoledronic acid, the examination of optical coherence tomography (OCT) revealed right macular edema with epiretinal membrane (arrow) and a normal left macula

Upon examination, her best-corrected visual acuity was 20/40 in the left eye and 20/60 in the right eye, and her intraocular pressure was 17 mm Hg, bilaterally. Slit-lamp examination revealed conjunctival hyperemia, ciliary injection, flare (2+), keratic precipitates (±), and trace cells in the anterior chamber. Posterior synechiae was also present. Fundus and optical coherence tomography (OCT) examinations revealed right macular edema (Fig. 1), and fluorescein angiography showed hyperfluorescence of the optic disk. Hematological exams of complete blood counts and transaminase levels were normal. The erythrocyte sedimentation was 95 mm/h, the C-reactive protein was elevated to 2.69 mg/dL, and the serum C4 level was slightly increased to 0.45 g/L. Bilateral acute uveitis was diagnosed and topical ganciclovir, pranoprofen, fluorometholone, and atropine were prescribed. Two days later, the ocular symptoms worsened, and treatment with retrobulbar injection of methylprednisolone and oral prednisolone was started. The patient’s ocular symptoms improved remarkably in the following 3 days, when best-corrected visual acuity reestablished to 20/30 bilaterally, and flare, keratic precipitates, cells, and macular edema disappeared. The patient continued with oral prednisolone, tapering slowly over the following 6 weeks. She achieved complete resolution, with no rechallenge and no recurrence at the 6 month follow-up.

## Discussion

According to the Naranjo adverse drug reaction probability scale, in our case study a score of 8 indicates a probable association between zoledronic acid and uveitis. Regarding previously published cases, a probable [10] to definite [5] causality has been reported.

The National Osteoporosis Foundation (NOF) recommends that any eye inflammation related to bisphosphonate should be reported to the healthcare provider as soon as possible [26]. However, due to the low prevalence and lack of recognition, although recommended by an ophthalmologist, the correct diagnosis was made 2 days after the onset of eye irritations, which worsened the ocular symptoms of our patient, and eye drops of antivirals were inappropriately prescribed. Fortunately, all the ocular manifestations recovered promptly, including the right macular edema.

Clinical features of 16 published cases (including our case) with ZAIU are shown in Table 1. Eight cases suffered from osteoporosis (including our case) [5–8, 13, 22, 23], four with bone metastasis from malignant tumors [9–11, 24], one with frontal hyperostosis and breast cancer [25], one with back and femur pain from the treatment of monoclonal gammopathy of undetermined significance (MGUS) [14], and the remaining two cases had risks of osteopenia due to leuprolide treatment for prostate cancer [12]. All the ocular manifestations occurred in 3 days. Only two out of 16 patients had a past ocular history. Bilateral eye involvement appeared in one-third of the patients, corresponding to a previous study [8]. The main ocular symptoms and signs included eye pain, blurred vision, diplopia, photophobia, lid edema, proptosis, conjunctival chemosis, hyperemia, and ophthalmoplegia. Nearly half of the patients presented with systemic symptoms. Posterior synechiae was observed in 6 patients, and most of the cases were cured. Choroidal folds [6] and vitreous haze [5] presented in some unusual cases. However, abnormal fundus was rarely reported, especially macular edema, as described in our case.

**Table 1:** Clinical features of the 16 published cases (including our case) with zoledronic acid induced uveitis

Case	Age	Eye involved	Time from exposure to onset of ocular menifestations	Indications	Ocular history
1 [5]	66	unilateral	2d	postmenopausal osteoporosis	no
2 [6]	75	unilateral	2d	osteoporosis	no
3 [7]	58	unilateral	NA	osteoporosis	no
4 [23]	58	unilateral	10 h	osteoporosis	no
5 [8]	56	unilateral	12 h	postmenopausal osteoporosis	no
6 [25]	70	unilateral	1d	frontal hyperostosis after breast cancer	no
7 [24]	54	bilateral	1d	breast cancer with bone matastasis	no
8 [9]	78	unilateral	48 h	prostate cancer with bone matastasis	no
9 [10]	48	unilateral	24 h	breast cancer with bone matastasis	no
10 [11]	54	unilateral	3d	breast cancer with lung and bone matastasis	no
11 [12]	56	bilateral	72 h	prostate cancer treated with leuprolide	no
12 [12]	68	bilateral	60 h	prostate cancer treated with leuprolide	no
13 [13]	60	unilateral	24 h	osteoporosis and breast cancer	yes
14 [14]	62	bilateral	48 h	back and femur pain in MGUS	no
15 [22]	59	unilateral	2d	osteoporosis	no
16^a^	63	bilateral	24 h	postmenopausal osteoporosis	yes

^a^The case described in this report, *NA* not available, *MGUS* monoclonal gammopathy of undetermined significance

Corticosteroids are usually necessary for treatment. Topical steroids and adequate treatment often lead in most cases to full recovery, but a small portion of patients only respond well to systemic use of steroids [8, 14]. No deterioration of osteoporosis has been reported to be related with steroid treatment.

Further use of bisphosphonates is not fully contraindicated. From our review of previous reports, in five patients given zoledronic acid with or without the protection of steroids, no additional ocular problems were reported [4, 12, 20]. Pamidronate was also prescribed to another patient, and a tolerance effect was also observed with a prior combination of steroids [27]. Interestingly, our patient developed uveitis soon after administration of intravenous zoledronate after a 2-year tolerance to oral alendronate. This is the first report of ZAIU after long-term alendronate tolerance.

Patel et al. retrospectively reported that 0.8 % of postmenopausal women with osteopenia receiving zoledronate developed mild to severe anterior acute uveitis [4]. More recently, the incidence of ZAIU was prospectively reported to be 1.1 % [95 % confidence interval (CI) 0.5–2.1] in a secondary analysis of a randomized controlled trial of early postmenopausal women [3]. Regarding oral bisphosphonates, a retrospective cohort study reported the incidence rate to be 29/10,000 person-years for uveitis in 10,827 first time users [28]. The true incidence might be higher, because some mild to moderate patients may fail to seek treatment.

APR is the most frequent adverse event after bisphosphonate use, usually including fever, fatigue, nasopharyngitis, musculoskeletal pain, and gastrointestinal symptoms. All APR components had a peak onset within 1 day, and the median duration of the APR was 3 days [21]. Recently, acute uveitis is being considered as part of APR after bisphosphonate dispensing, due to its similar time of occurrence [4, 8]. Bisphosphonates have been shown to share homologies with γ/δ T-cell ligands by stimulating cytokine release by γ/δ lymphocytes, and by inhibiting farnesyl diphosphate synthase to increase the intracellular accumulation of isopentenyl pyrophosphate [29]. These activities may contribute to the development of APR and uveitis.

Reid et al. investigated 7,765 postmenopausal women with osteoporosis, using a stepwise logistic regression analysis, and showed that APRs were less common in smokers, diabetics, calcitonin users, and previous oral bisphosphonate users [21]. Because uveitis is a part of APR, it seems to develop less frequently after oral bisphosphonate tolerance, as described in our case study. Additional and larger case studies and single case reports are still needed to confirm the risk factors of ZAIU after previous bisphosphonate use.

## Conclusions

We report a case of ZAIU with macular edema after long-term oral alendronate tolerance. ZAIU is beginning to be accepted as part of APR due to the similar time of occurrence. Endocrinologists and ophthalmologists should be aware of this drug reaction, in cases of deterioration of ocular symptoms and the inappropriate use of antibiotics and antiviral agents. Prior oral alendronate may not entirely prevent ZAIU. Steroids are usually necessary in the treatment of ZAIU, and systemic steroids are sometimes indicated. The readministration of bisphosphonate is not fully contraindicated, but according to the available evidence prior steroid protection may be a more reasonable treatment strategy.

### Consent

Written informed consent was obtained from the patient for publication of this case report and any accompanying images. A copy of the written consent is available for review by the Editor of this journal.

## Abbreviations

OCT: Optical coherence tomography
ZAIU: Zoledronic acid induced uveitis
APR: Acute phase reaction
MGUS: Monoclonal gammopathy of undetermined significance
NOF: National Osteoporosis Foundation
